# Risk Identification of Bronchopulmonary Dysplasia in Premature Infants Based on Machine Learning

**DOI:** 10.3389/fped.2021.719352

**Published:** 2021-08-17

**Authors:** Jintao Lei, Tiankai Sun, Yongjiang Jiang, Ping Wu, Jinjian Fu, Tao Zhang, Eric McGrath

**Affiliations:** ^1^School of Science, Guangxi University of Science and Technology, Liuzhou, China; ^2^Department of Neonatology, Liuzhou Maternity and Child Health Care Hospital, Liuzhou, China; ^3^Department of Pharmacy, Chengdu First People's Hospital Chengdu Integrated TCM Western Medicine Hospital, Chengdu, China; ^4^Department of Preventive Medicine, Liuzhou Maternity and Child Health Care Hospital, Liuzhou, China; ^5^Children's Hospital of Michigan, Wayne State University School of Medicine, Detroit, MI, United States

**Keywords:** premature infants, bronchopulmonary dysplasia, feature selection, risk identification, machine learning

## Abstract

Bronchopulmonary dysplasia (BPD) is one of the most common complications in premature infants. This disease is caused by long-time use of supplemental oxygen, which seriously affects the lung function of the child and imposes a heavy burden on the family and society. This research aims to adopt the method of ensemble learning in machine learning, combining the Boruta algorithm and the random forest algorithm to determine the predictors of premature infants with BPD and establish a predictive model to help clinicians to conduct an optimal treatment plan. Data were collected from clinical records of 996 premature infants treated in the neonatology department of Liuzhou Maternal and Child Health Hospital in Western China. In this study, premature infants with congenital anomaly, premature infants who died, and premature infants with incomplete data before the diagnosis of BPD were excluded from the data set. After exclusion, we included 648 premature infants in the study. The Boruta algorithm and 10-fold cross-validation were used for feature selection in this study. Six variables were finally selected from the 26 variables, and the random forest model was established. The area under the curve (AUC) of the model was as high as 0.929 with excellent predictive performance. The use of machine learning methods can help clinicians predict the disease so as to formulate the best treatment plan.

## Introduction

In recent decades, the survival rate of extremely premature infants has been increased by the advances in newborn care, but the incidence of newborns is still high (1, 2). Bronchopulmonary dysplasia (BPD) is the most common disease in preterm infants. Most surviving children with BPD also have obstructive airway disease, pulmonary hypertension, and growth retardation, which seriously threaten the safety and quality of life of premature infants and impose a serious burden on the family and society (3). The diagnosis and classification of BPD are mainly based on the standards published by the National Institute of Child Health and Human Development (NICHD) in 2001. For premature infants born at a gestational age of <32 weeks, BPD is the cumulative use of supplemental oxygen for 28 days or more, and then the need for no oxygen inhalation, oxygen inhalation at a concentration <0.30, and oxygen inhalation at a concentration of 0.30 or positive pressure ventilation and mechanical ventilation at 36 weeks of gestational age is respectively classified as mild, moderate, and severe BPD (sBPD) (4). The diagnosis of BPD in very preterm infants has been updated by Jensen et al. in the *American Journal of Respiratory and Critical Care Medicine*. Their study defined BPD according to treatment with the following support at 36 weeks' postmenstrual age, regardless of prior or current oxygen therapy: no BPD, no support; grade 1, nasal cannula ≤ 2 L/min; grade 2, nasal cannula > 2 L/min or noninvasive positive airway pressure; and grade 3, invasive mechanical ventilation (5).

In existing studies, most research used the logistic regression method. The study by Laughon et al. based on multi-class variables (death, severe BPD, moderate BPD, mild BPD, and no BPD) developed and validated models for BPD risk at six postnatal ages using gestational age, birth weight, race and ethnicity, gender, respiratory support, and fraction of inspiration O_2_ (FIO_2_) (6). Some studies of the two-class result include having BPD vs. no BPD or having BPD/death against no BPD. Valenzuela-Stutman et al. found that the important factors affecting BPD are birth weight, gestational age, and ventilation duration (7). The results of the study of Ding et al. revealed that the combination of sB7-H3 (day 7), IL-18 (day 14), NCIS, and clinical risk factors (electrolyte disturbances, hs-PDA, and the age that infants achieved 120 kcal/kg·day via enteral feeding ≥40 days after birth) might serve as an optimal predictive model for the occurrence of BPD (8). Bentsen et al. added the flow data of the ventilator to calculate the flow parameters in the study. The TEF50/PTEF parameter also revealed good predictive ability in this respect. The model that combines this parameter with the birth weight *z* score and gender can accurately predict advanced respiratory disease (9). Morrow et al. put some maternal features into the model and found out that smoking and high blood pressure increase the risk of BPD after preterm delivery and that maternal smoking is closely related to the increased risk of early and late childhood respiratory diseases (10).

Besides the logistic regression belonging to the traditional statistical method, some studies based on machine learning also had excellent results. Ambalavanan et al. discovered that risk factors were related to low birth weight, high oxygen demand, male sex, administration of an extra surfactant dose, high oxygen index, and birth status but not related to alveolar oxygen partial pressure (PaO_2_) and inhaled nitric oxide (iNO) via a decision tree (11). In addition, Verder et al. added spectral data to the model, using support vector machine, which predicted BPD with spectral data (12). However, the scale of the data was too small.

As mentioned previously, a problem in previous studies is the insufficient sample size. Due to the insufficiency of samples, some studies with the ordinary linear model do not even set up the training set and testing set to evaluate the generalizability of the model. The result may look exciting, but technically, it may be the result of over-fitting. In today's era, when big data and machine learning are popular, researchers prefer to use large capacity data for analysis. In addition, the traditional method of using logistic regression to retain variables whose regression coefficient *p*-value is less than the critical value of 0.05 is commonly used, but there are still some algorithms that can be used in feature selection and predictions, which the current work on this disease lacks. Results obtained by some researchers indicate that oxygen inhalation time is a risk factor for BPD, which is obvious according to the criteria for BPD. Researchers should strive to find other clinical predictors and obtain more comprehensive indicators for better prediction. Therefore, in order to improve on the above-mentioned problems, this paper collects data with a large sample size based on the results of previous studies and expert consensus and adopts the method of ensemble learning in machine learning, using the Boruta algorithm with the random forest algorithm.

## Data and Methods

### Object

This is a retrospective study from data collected from clinical records of 996 premature infants treated in the neonatology department of Liuzhou Maternal and Child Health Hospital in Western China. All data were de-identified.

### Ethical Statements

The research design and operation process of the project have been approved by the ethics committee of Liuzhou Maternal and Child Health Hospital with a waiver for informed consent (approval number: 201900513) due to the retrospective nature of this chart review.

### Involvement of Patients and the Public

Due to its retrospective nature, this cohort study did not involve patients and the public in the design or conduct or reporting or dissemination plans.

### Data

In this study, premature infants with congenital anomaly, premature infants who died, and premature infants with incomplete data before the diagnosis of BPD were excluded from the data set. After exclusion, we included 648 premature infants in the study, 149 premature infants with BPD and 499 premature infants without BPD. The definition of premature infant was any infant born at a gestational age <37 weeks. BPD diagnosis and classification standards were inferred from the those established by the NICHD/National Heart, Lung, and Blood Institute (NHLBI).

The data set in this article provides a rich collection of information about the state of the mother and the early state of the newborn. The predictors are selected according to the consensus of experts, including sex, maternal age, gestational age, multiple births, premature rupture of the membrane, intrauterine distress, antenatal corticosteroid, maternal infection, pregnancy complications, normal delivery, gross birth weight, Apgar 1 (A1), Apgar 5 (A5), respiratory distress syndrome (RDS), septicemia, anemia, pulmonary hemorrhage, patent ductus arteriosus (PDA), patent foramen ovale (PFO), phototherapy, respiratory support, duration of oxygen therapy (unit: min), surfactant, first partial pressure of carbon dioxide (first PCO_2_, unit: mmHg), first mean airway pressure (first MAP, unit: cm H_2_O), and first FIO_2_ (unit: mmHg). Mean and standard deviation of continuous variables are shown in Table 1. First MAP, first PCO_2_, and first FIO_2_ represent the first measurement of these three variables at the time of initiating ventilatory support. The Kolmogorov–Smirnov test was performed to test if they obeyed normal distribution. Independent *t*-tests were performed for variables that obey normal distribution, and Mann–Whitney *U*-tests for others. Categorical variables are presented as frequencies with percentages in Table 1 and analyzed by chi-square test or Fisher's exact test, as appropriate. A *p* < 0.05 is considered to be statistically significant, as shown in Table 1, for the clinical characteristics of BPD. It is noteworthy that respiratory support was given to 266 infants in the no-BPD group. In other words, the observed values of first FIO_2_ of the other 233 infants in the no-BPD group are 0. Therefore, the mean of first FIO_2_ in the no-BPD group is lower than that of room air (21%).

**Table 1:** Clinical characteristics by BPD status.

**Variable**	**With BPD** **(*n* = 149)**	**Without BPD** **(*n* = 499)**	***p*-value**
Gender (males), *n* (%)	92 (61.7%)	242 (48.5%)	0.005
Maternal age, mean (SD)	31 (5.72)	29 (5.73)	<0.001
Gestational age (weeks), mean (SD)	28 (2.42)	30 (2.20)	<0.001
Multiple births, *n* (%)	50 (34%)	194 (39%)	0.239
PROM, *n* (%)	52 (35%)	170 (34%)	0.851
Intrauterine distress, *n* (%)	15 (10%)	92 (18%)	0.016
Antenatal corticosteroid, *n* (%)	88 (59%)	290 (58%)	0.837
Maternal infection, *n* (%)	19 (13%)	56 (11%)	0.609
Pregnancy complications, *n* (%)	39 (26%)	138 (28%)	0.722
Normal delivery, *n* (%)	103 (69%)	3,230 (46%)	<0.001
Gross birth weight (g), mean (SD)	1,094 (195.07)	1,236 (195.36)	<0.001
A1, mean (SD)	8 (2.36)	9 (2.36)	<0.001
A5, mean (SD)	9 (1.51)	9 (1.51)	<0.001
RDS, *n* (%)	84 (56%)	133 (27%)	<0.001
Septicemia, *n* (%)	30 (20%)	82 (16%)	0.294
Anemia, *n* (%)	131 (88%)	324 (65%)	<0.001
Pulmonary hemorrhage, *n* (%)	13 (6%)	31 (4%)	0.179
PDA, *n* (%)	3 (1%)	2 (1%)	0.325
PFO, *n* (%)	50 (34%)	111 (22%)	0.005
Phototherapy, *n* (%)	109 (73%)	315 (63%)	0.024
Respiratory support, *n* (%)	141 (95%)	269 (54%)	<0.001
Duration of oxygen therapy (h)	1,051 (526.20)	187 (526.77)	<0.001
Surfactant, *n* (%)	91 (61%)	127 (26%)	<0.001
First PCO_2_ (mmHg), mean (SD)	47 (25.66)	25 (25.67)	<0.001
First MAP (cm H_2_O), mean (SD)	9 (4.59)	5 (4.59)	<0.001
First FIO_2_ (%), mean (SD)	39 (23.35)	20 (23.37)	<0.001

*PROM, premature rupture of membranes; A1, 1 min Apgar score; A5, 5 min Apgar score; RDS, respiratory distress syndrome; PDA, patent ductus arteriosus; PFO, patent foramen ovale; First PCO_2_, first measurement of partial pressure of carbon dioxide; First MAP, first measurement of mean airway pressure; First FIO_2_, first measurement of fraction of inspiration O_2_*.

### Statistical Methods

Feature selection is usually an important step in the application of machine learning methods. Generally speaking, the current popular feature extraction method is mainly used to extract nonredundant features, which is a minimal–optimal problem (13), and the Boruta algorithm is dedicated to the problem of extracting all-relevant features with the response variable. This is of necessity for medical problems. The Boruta algorithm creates a random forest model in each iteration by duplicating all features and shuffling them in order and calculates the *z* score of each attribute. The *z* score is calculated by dividing the average loss by its standard deviation, which can be used as an important measurement. Find the largest *z* value in the shadow attributes and mark attributes whose *z* values are higher than this value a hit until all attributes are marked or reach the end of the random forest running limit. Through this process, the features that are most relevant to the response variable are selected. The Boruta algorithm can reduce the misleading effects of random fluctuations and correlations by increasing the randomness of the system and collecting results from random sample sets. This extra randomness will give a clearer understanding of which attributes are really important (14).

We initially used all 26 variables including maternal and infantile features as predictors. Note that the predictive performance of the model may not be more accurate with more predictors. The more variables there are, the higher the complexity of the model could be, affecting the prediction results and running speed. Usually, a small subset is sufficient to obtain a strong prediction performance. Therefore, we used the Boruta algorithm with 10-fold cross-validation for feature selection in our research. After the selection is completed, a random forest model was established.

As one of the classic algorithms of machine learning, random forest has high accuracy in disease risk prediction and diagnosis. At present, machine learning methods have been successfully used in the diagnosis and prognosis of various diseases, such as in predicting stroke and intracranial hemorrhage (15), but they are not widely used in premature infant studies. Random forest is an integrated learning method based on decision trees, including multiple decision trees generated randomly and outputting their predictions. Random forest adopts bagging thought and characteristic subspace, which has better noise resistance than a single decision tree; is not easy to over-fit; and improves the generalization ability significantly (16). The number of decision trees constructed in this study was 500, and three variables were randomly selected on each decision tree node. Random forest selects or excludes variables based on the importance of features. A simplified model is established based on the selected variable, rather than a complete model that includes all variables. Then the model is verified in the testing set, using the following parameters as evaluation tools: area under the curve (AUC)/C-static, accuracy, sensitivity, specificity, positive prediction rate, and negative prediction rate.

In this study, R software (version 4.0.2) was used for data analysis. R packages “caret,” “Boruta,” “randomForest,” and “pROC” are used to develop and verify the Boruta and random forest models.

## Results

After inclusion and exclusion from January 2017 to September 2020, a total of 648 preterm newborns met the inclusion criteria. Among them, the number of neonatal weeks ranged from 24 to 36 weeks. Before establishing the model, the data set was divided into training and testing sets at random, with a ratio of 85:15. We used the createDataPartition() function from “caret” package in the R to perform mixed stratified random sampling for the classification labels, so as to ensure that the distribution proportions of the various labels in the training set and the testing set and the distribution proportion of the sample population are unanimous. The training set contains 552 infants, among which 127 infants had BPD and 425 infants had no BPD. The testing test contains 96 infants, among which 74 infants had BPD and 22 infants had no BPD.

**Figure 1:**
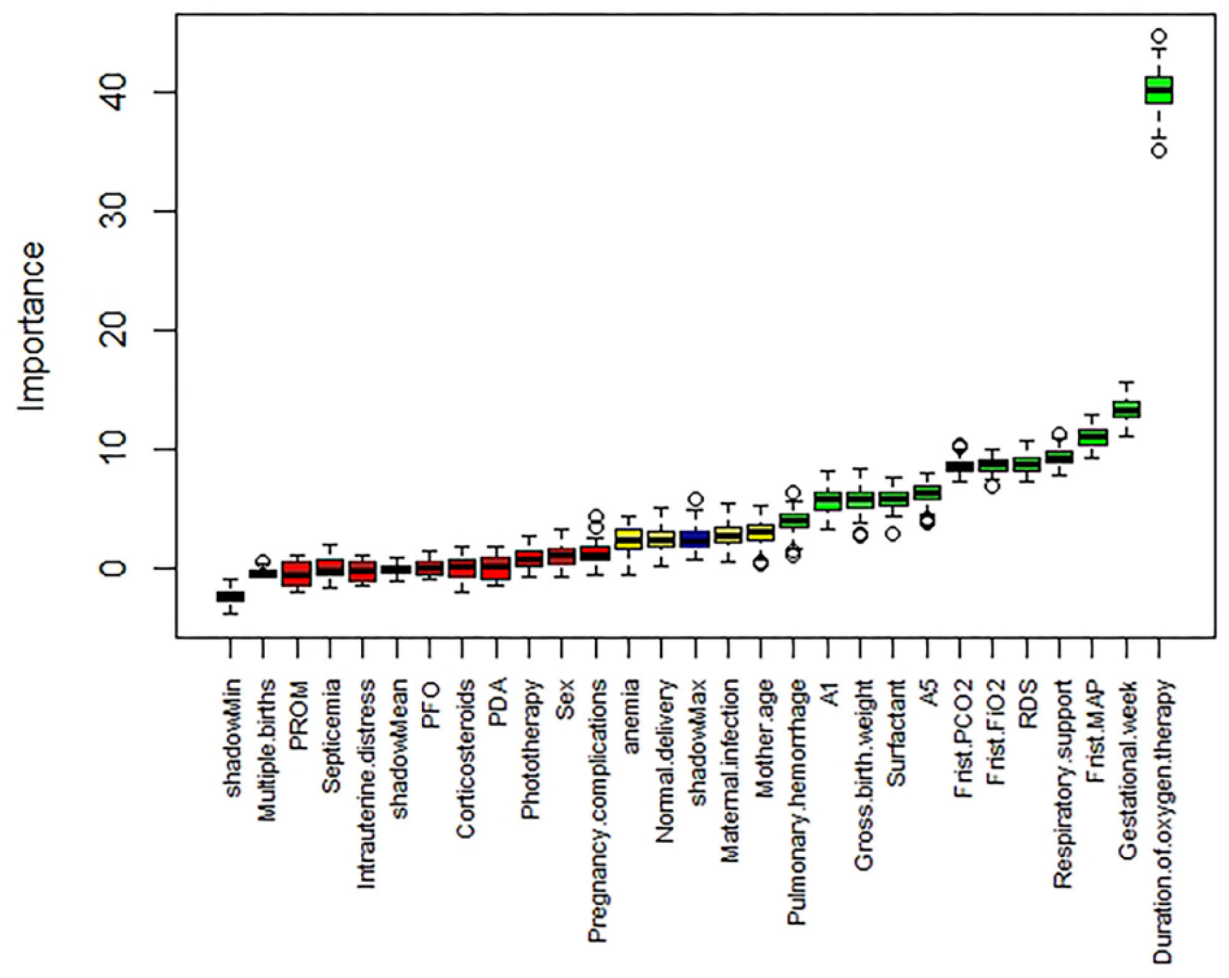
Boruta screening feature results.

First, the Boruta feature screening is performed on the training set. After 100 iterations, the result is shown in Figure 1. The horizontal axis is the name of each variable, and the vertical axis is the *z*-score of the variable. The box plot depicts the *z*-score of each variable during model calculation. The green boxes represent the first 12 important variables. The yellow represents tentative attributes, and the red represents unimportant variables. The top 12 variables in descending order of *z*-score were duration of oxygen therapy, gestational age, first MAP, respiratory support, RDS, first FIO_2_, first PCO_2_, A5, surfactant, gross birth weight, A1, and pulmonary hemorrhage.

After the feature selection, a random forest model recorded as model 1 on the training set is deployed. The receiver operating characteristic (ROC) curve plotted in blue in **Figure 3** shows the performance of model 1 on the testing set with a fabulous AUC of 0.922, which implies that the model has a desirable classification performance. The threshold of 0.455 is confirmed according to the Youden index (YI) (17). Since the predicted value of the corresponding variable is a real value between 0 and 1, the case with a predicted value greater than 0.455 is classified as a positive case (BPD); otherwise, it is classified as a negative category (no BPD). The confusion matrix of the testing set and relevant indicators are shown in Table 2. The accuracy value of the model reached 0.9167, and it was believed that the model had relatively good performance.

**Table 2:** Confusion matrix and evaluation indicators of model 1 on the testing set.

**Prediction**		**Actual**	
	0		1
0	70		4
1	4		18
Accuracy		0.9167	
Precision		0.9459	
Sensitivity		0.8182	
Specificity		0.9459	

**Figure 2:**
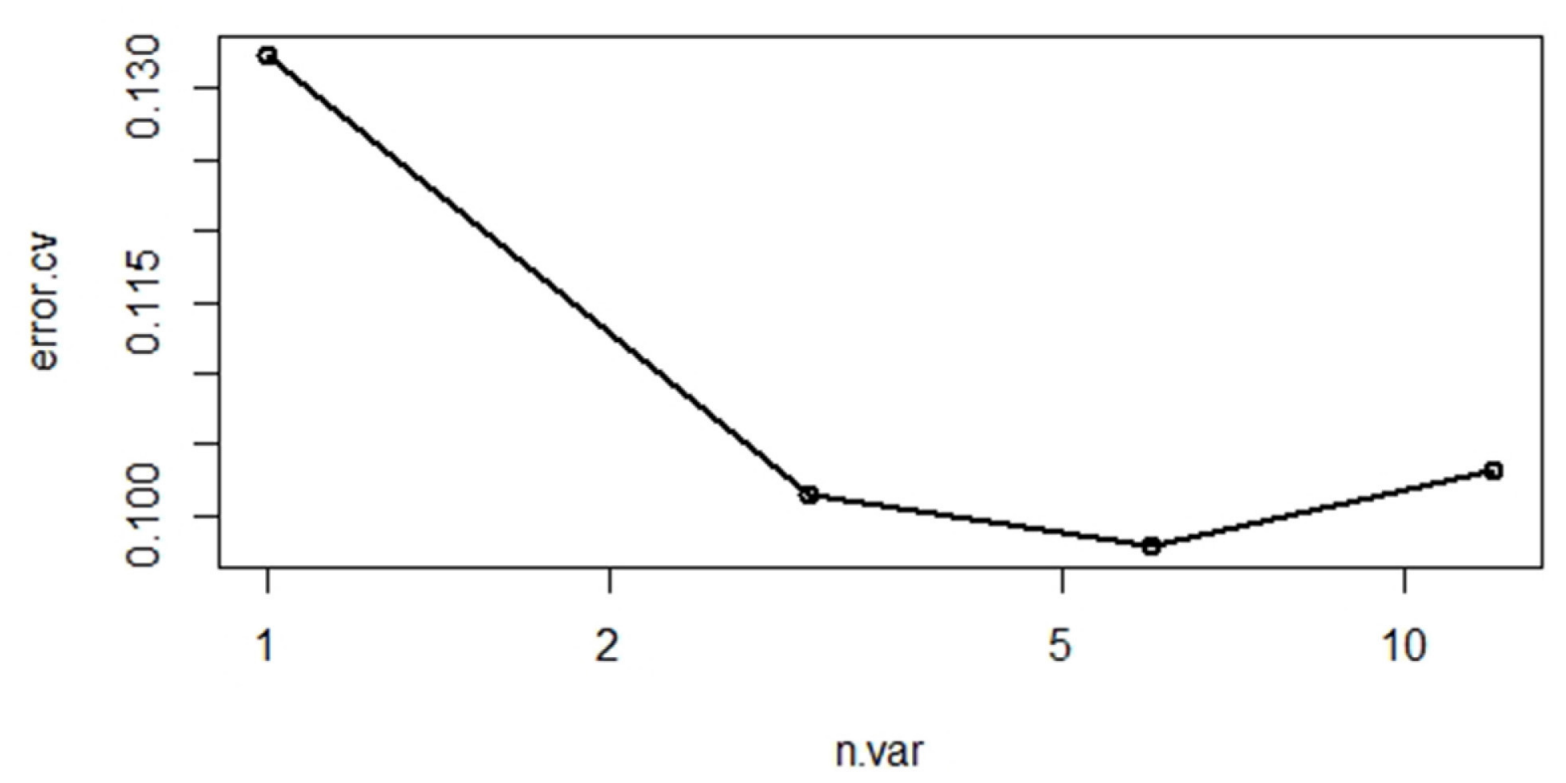
Cross-validation results.

**Figure 3:**
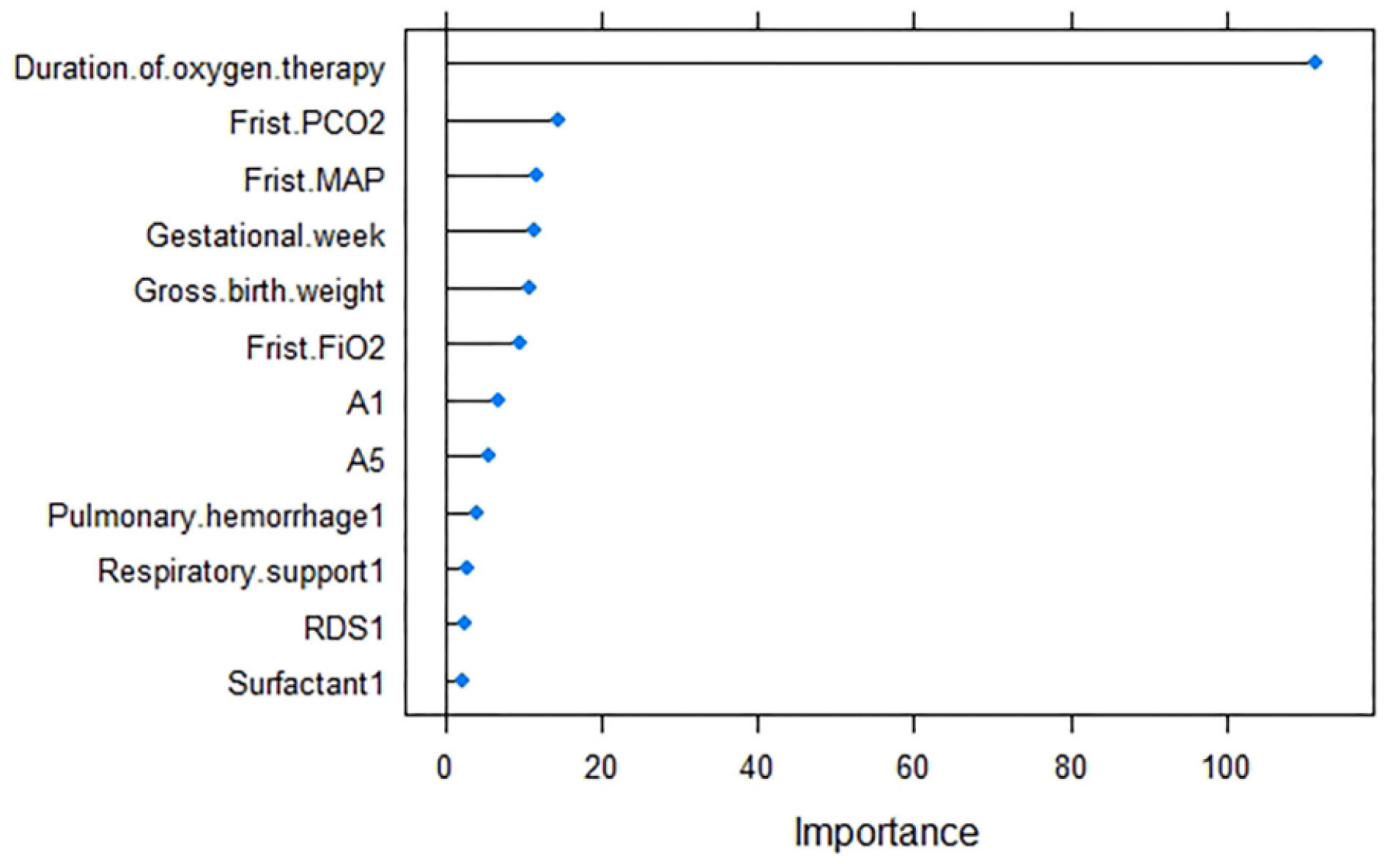
Model 1 variable importance.

However, even though model 1 performed well, 12 variables seem to be too many from a clinical point of view. In order to pursue simplicity, a simpler model could be found by reducing the number of features while ensuring the performance of the model simultaneously. The number of variables with the smallest error in the random forest model algorithm is obtained through 10-fold cross-validation. Figure 2 shows that when the number of variables is 6, the cross-validation error reaches the minimum. Figure 3 shows the rank of importance of 12 variables in model 1. Therefore, six final variables were selected: total oxygen inhalation time, first PCO_2_, first MAP, gestational age, gross birth weight, and first FIO_2_. The random forest model recorded as model 2 was established again for the above variables.

**Figure 4:**
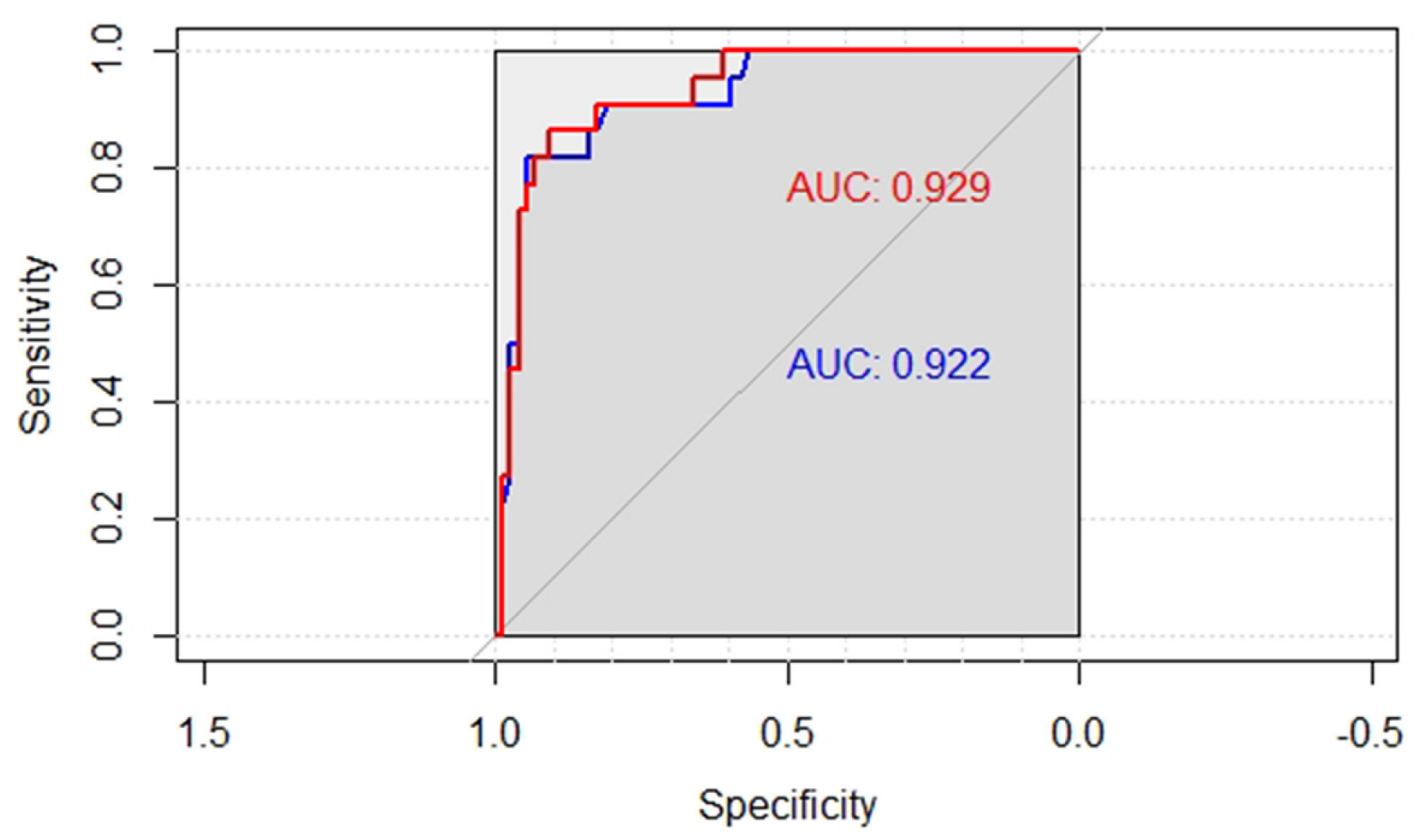
ROC curves of the two models on the testing set.

The ROC curve of model 2 on the same testing set is shown in Figure 4, plotted in red. It is worth mentioning that model 2 is simpler than model 1, but the AUC value of 0.929 is slightly higher than that in model 1. Although this difference is not obvious, it can be considered that the performance of model 2 is consistent with that of model 1. The ROC curve shows that the threshold value should be 0.43. So similar to the process of model 1 above, the case with a predicted value greater than 0.287 was classified as a positive case (BPD); otherwise, it was classified as a negative category (no BPD). The confusion matrix and performance indicators are shown in Table 3. The accuracy of model 2 reached 0.8958.

**Table 3:** Confusion matrix and evaluation indicators of model 2 on the testing set.

**Prediction**		**Actual**	
	0		1
0	63		6
1	7		25
Accuracy		0.8958	
Precision		0.8636	
Sensitivity		0.7308	
Specificity		0.9571	

**Figure 5:**
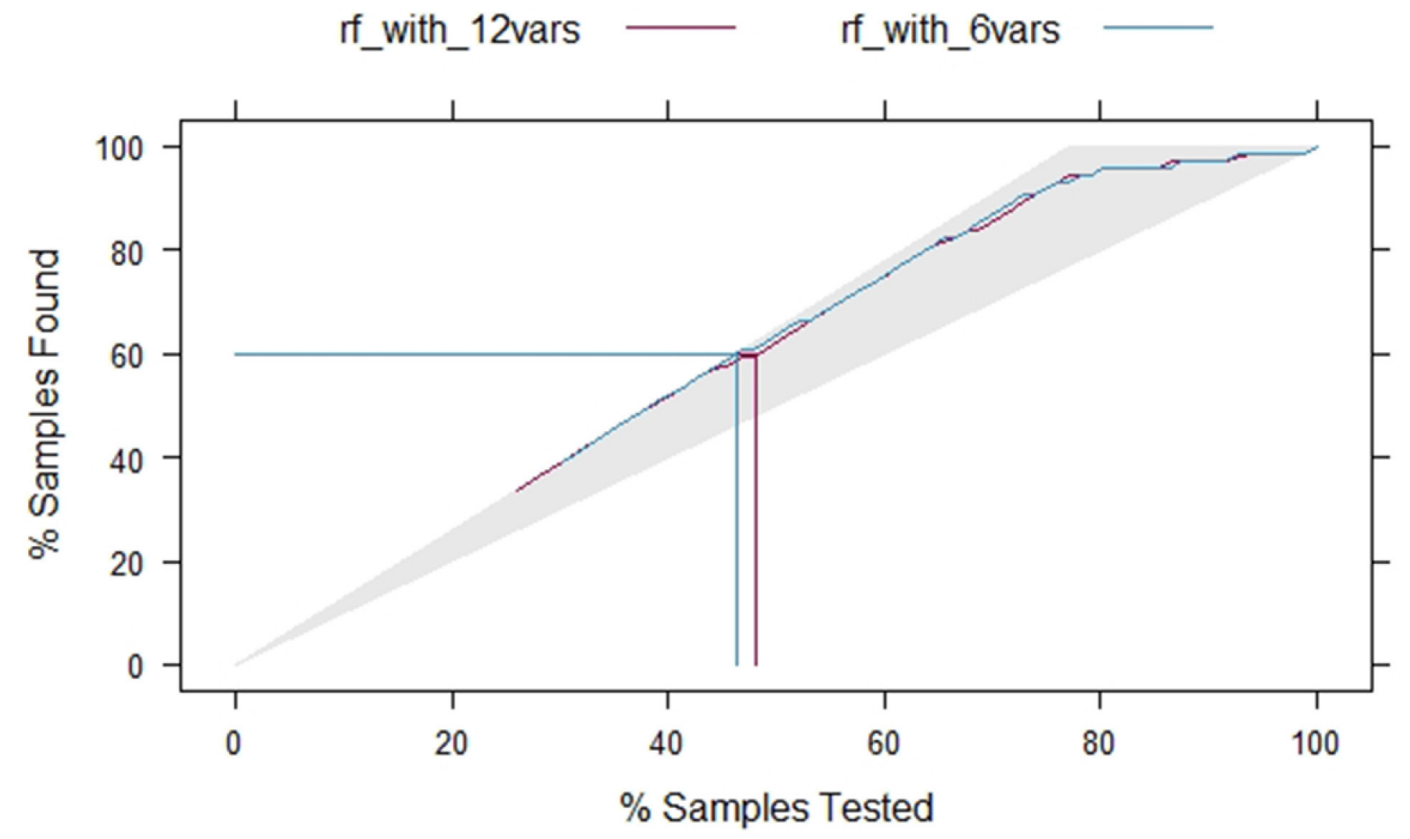
Lifting curves of the two models.

If the ROC curve of a model can completely cover another, it means that the model has a significant advantage. Figure 4 shows two ROC curves. The blue curve is the ROC curve of model 1, and the red curve is that of model 2. The ROC curve of model 2 (with a higher AUC) fails to cover the other curve completely, indicating that there is no significant performance difference between the two models. In addition, a lift chart can also compare the performance of the two models. Figure 5 shows the lifting diagrams of the two models. Similarly, there is no case where one of the two curves completely envelopes the other.

Therefore, both model 1 and model 2 can be used to predict BPD. However, model 2 is simpler than model 1, with less features and a still good performance. A smaller number of clinical features are easier to obtain accurately. Model 2 was selected as the final prediction model.

## Discussion

At present, the clinical cause and pathogenesis of BPD have not yet been fully clarified. It is generally believed that it is related to lung injury caused by genetics, oxygen toxicity, infection, mechanical ventilation, inflammation, and abnormal repair after injury. During the occurrence and development of BPD, the original underlying diseases, complications, oxygen therapy, and invasive ventilation of premature infants all play a vital role. There is no clinically effective method to cure BPD, so the clinic attaches great importance to the screening and prevention of BPD in high-risk children.

To clarify the external risk factors of children with BPD and make early predictions, this study first selected BPD preterm infants and no-BPD preterm infants for a controlled study. The results showed that maternal age, gestational age (weeks), normal delivery, gross birth weight, A1, A5, RDS, anemia, respiratory support, duration of oxygen therapy, surfactant administration, first PCO_2_, and first MAP factor had *p* < 0.05 with statistical significance. From there, machine learning methods may be employed to provide empirical evidence for early prediction of BPD on large batches of data. To this end, we developed a computational model for early prediction of BPD; through the combination of feature screening and random forest, an auxiliary diagnosis system for BPD is finally constructed. The model is trained on a large data set containing 26 clinical experiment variables; only six inputs are enough to achieve a strong predictive performance in the end; the AUC of the model in the testing set is as high as 0.929. In the future, we will export the model as a tool to assist in predicting BPD and will be further verified in external data testing.

Although the main goal of machine learning models is usually to predict, this study also reveals the risk factors that affect BPD in preterm infants. It was found that total oxygen inhalation time, first PCO_2_, first MAP, gestational age, gross birth weight, and first FIO_2_ were the most important predictors in the model. Compared with the 26 variables in the previous study, only a small part of the variables provided by most hospitals are needed. Therefore, the resultant model is more generally applicable to the clinical environment and has a positive significance in saving the lives of premature infants. The important features of this study are consistent with previous studies to a certain extent. Low gestational age and low birth weight, which may lead to immaturity, are risk factors for BPD. The most important feature is the duration of oxygen therapy, for the definition of BPD is based on oxygen dependence. Thus, pediatricians should take care of those infants who need long-time ventilatory support. Research uses segmentation like mechanical ventilation for >7 days as a predictor for outcome of BPD (7). In a further study, a model of risk scores in various duration of mechanical ventilation can be investigated with sufficient data collected on different time periods.

There are some limitations in this study. (1) The patient population of the study is not representative of typical infants with BPD. Most of the infants who develop BPD are <28 weeks of gestational age and <1,000 g in birth weight. In this study, the gestational ages of 56 infants are greater than 28 weeks in the BPD group (62% in BPD group). The birth weights of 112 infants are greater than 1,000 g in the BPD group (75% in the BPD group). (2) The numbers of premature infants with and without BPD do not match, which may reduce the generalization of the model. (3) The lack of standardization of data in the collection of original data may also be a limitation as it may reduce the calculation accuracy.

The random forest model in this study had an overall good performance. In future work, we will plan to test other machine learning algorithms (such as KNN, SVM, and CART). We expect that the model developed will help neonatologists to identify infants at risk of BPD in a timely manner in the future and in this way possibly help clinicians to reduce the incidence and chronic nature of BPD.

## Conclusion

The thesis finally confirmed six variables for the establishment of the random forest model after obtaining 26 variables through feature engineering. The model which could help clinicians to make the best treatment plan for patients with BPD presented a relatively good performance for prediction, with an AUC of 0.929. In future research, researchers can use machine learning algorithms to develop more disease prediction models to assist treatment.

## Data Availability Statement

The original contributions presented in the study are included in the article/supplementary material, further inquiries can be directed to the corresponding author/s.

## Ethics Statement

The studies involving human participants were reviewed and approved by The Ethics Committee of Liuzhou Maternal and Child Health Hospital. Written informed consent to participate in this study was provided by the participants' legal guardian/next of kin.

## Author Contributions

TZ, JF, JL, TS, YJ, PW, and EM: study conceptualization. TZ, JF, TS, and JL: data curation. JL and TS: formal analysis, data interpretation, data visualization, and writing original draft. TZ and JF: funding acquisition for study and project administration. TZ, JF, JL, TS, YJ, and PW: methodology. TZ, JF, YJ, PW, and EM: supervision. JL, TS, and EM: writing, critical intellectual input, and review. All authors contributed to the article and approved the submitted version.

## Conflict of Interest

The authors declare that the research was conducted in the absence of any commercial or financial relationships that could be construed as a potential conflict of interest.

## Publisher's Note

All claims expressed in this article are solely those of the authors and do not necessarily represent those of their affiliated organizations, or those of the publisher, the editors and the reviewers. Any product that may be evaluated in this article, or claim that may be made by its manufacturer, is not guaranteed or endorsed by the publisher.
